# Budget constrained machine learning for early prediction of adverse outcomes for COVID-19 patients

**DOI:** 10.1038/s41598-021-98071-z

**Published:** 2021-10-01

**Authors:** Sam Nguyen, Ryan Chan, Jose Cadena, Braden Soper, Paul Kiszka, Lucas Womack, Mark Work, Joan M. Duggan, Steven T. Haller, Jennifer A. Hanrahan, David J. Kennedy, Deepa Mukundan, Priyadip Ray

**Affiliations:** 1 Lawrence Livermore National Laboratory, 7000 East Ave, Livermore, CA 94550 USA; 2 ProMedica Health System, Inc, 3103 Executive Pkwy, Toledo, OH 43606 USA; 3 Department of Medicine, University of Toledo College of Medicine and Life Sciences, 3000 Arlington Ave, Toledo, OH 43614 USA; 4 Department of Pediatrics, University of Toledo College of Medicine and Life Sciences, 3000 Arlington Ave, Toledo, OH 43614 USA

**Keywords:** Outcomes research, Computational science

## Abstract

The combination of machine learning (ML) and electronic health records (EHR) data may be able to improve outcomes of hospitalized COVID-19 patients through improved risk stratification and patient outcome prediction. However, in resource constrained environments the clinical utility of such data-driven predictive tools may be limited by the cost or unavailability of certain laboratory tests. We leveraged EHR data to develop an ML-based tool for predicting adverse outcomes that optimizes clinical utility under a given cost structure. We further gained insights into the decision-making process of the ML models through an explainable AI tool. This cohort study was performed using deidentified EHR data from COVID-19 patients from ProMedica Health System in northwest Ohio and southeastern Michigan. We tested the performance of various ML approaches for predicting either increasing ventilatory support or mortality. We performed post hoc analysis to obtain optimal feature sets under various budget constraints. We demonstrate that it is possible to achieve a significant reduction in cost at the expense of a small reduction in predictive performance. For example, when predicting ventilation, it is possible to achieve a 43% reduction in cost with only a 3% reduction in performance. Similarly, when predicting mortality, it is possible to achieve a 50% reduction in cost with only a 1% reduction in performance. This study presents a quick, accurate, and cost-effective method to evaluate risk of deterioration for patients with SARS-CoV-2 infection at the time of clinical evaluation.

## Introduction

Since the outbreak of coronavirus disease 2019 (COVID-19) in the United States in early 2020^1^, many hospitals and clinics have experienced shortages of ventilators and bed spaces in Intensive Care Units (ICUs)^2^. At the first encounter with a COVID-19 patient, as evidenced by a positive nasopharyngeal PCR test for SARS-CoV-2, it is critical to determine using readily available clinical information whether hospitalization is required, or outpatient treatment can be utilized without risk of deterioration or increased morbidity and mortality. With the increasing availability of electronic health records (EHRs) of hospitalized COVID-19 patients, data-driven decision support systems, such as those based on Machine Learning (ML) methodologies, have been explored extensively in the recent literature as a means of triaging patients with COVID-19 at the point of contact with the health care system^3–6^. However, for wide-spread adoption of such ML systems, two fundamental challenges remain. First, to gain the confidence of health care providers, clinical interpretability of the ML algorithms is crucial. Second, the cost and availability of various laboratory tests can vary substantially across facilities and geographic locations. Hence, accounting for general test availability and costs in the ML decision tool is critical.

Interpretability of ML algorithms for clinical applications has recently received significant research attention^7–10^, as the lack of interpretability can potentially have adverse or even life-threatening consequences. In general, there exists a trade-off between an ML model’s predictive performance and interpretability: Linear models are highly interpretable, but they may not have enough capacity to capture the complexity of EHR data, whereas non-linear models typically provide better predictive performance, but they can be hard to interpret. In Lundberg and Lee^11^, the authors introduce SHAP, a software package that leverages the game-theoretic concept of Shapley values to explain the output of any ML model. Shapley plots have recently been used in ML applications to risk assessment of COVID-19 patients^12, 13^.

While recent studies have considered the interpretability of ML algorithms that triage COVID-19 patients based on clinical features, *the availability and cost of such clinical features have largely been ignored*. However, this is an important consideration, since many hospitals reached near full capacity at the peak of the pandemic, bringing the economic sustainability and ethicality of resource allocation of the healthcare system into question. Moreover, recent studies have found that patients are often over-diagnosed by unnecessary testing services, which may delay care for those patients who have more immediate need for medical attention. This suggests that taking the cost of diagnostic testing into account when building ML decision support tools can not only help satisfy budget constraints in resource-constrained environments, but it can also lead to better patient-centered outcomes.

Machine learning under budget constraints is also known as *cost-sensitive* or *cost-constrained learning*. This field is focused on developing good predictive models while also taking into consideration the cost of collecting data. Several works have considered the general problem of cost-sensitive feature selection in machine learning. These works have tended to provide approximate methods or heuristics for solving the cost-constrained optimization problems associated with such tasks^14–17^. In healthcare applications, a number of previous works have introduced budget constraints, such as the financial costs of lab tests or clinical preferences, into their proposed machine learning models^18, 19^. However, to the best of our knowledge, *our work is the first to consider cost-sensitive learning applied to COVID-19*. In this paper, we propose an interpretable ML framework that includes the consideration of the financial cost of clinical features. More specifically, we sought to (1) develop an ML approach for predicting adverse outcomes based on demographic information, co-morbidities, and biomarkers collected close to the date of a positive PCR test; (2) gain insight into the decision-making process of the ML algorithm based on interpretable ML tools, such as Shapley values; and (3) identify and account for unequal costs of clinical features in the ML decision support system by finding the optimal set of biomarkers that results in the highest utility under a given cost structure.

A retrospective study was performed using EHRs from patients in the largest health care system in northwestern Ohio and southeastern Michigan (ProMedica Health System). The data used in this study corresponds to patients who (1) had a positive nasopharyngeal PCR test for SARS-CoV-2 between March 20, 2020 and December 29, 2020, and (2) were admitted to the hospital shortly before or after the positive result. Demographics (e.g., age, race, co-morbidities, insurance status), vitals, and a wide range of lab tests available within 3 days of a first positive PCR test were used to train machine learning models to predict a patient’s risk of adverse outcomes, namely, *composite ventilation* and *mortality*. Our studies indicate that, compared to a baseline linear logistic regression model, more advanced nonlinear or tree-based classifiers provide improved performance both in terms of average precision (AP) and the area under the receiver operating characteristic curve (AUC). The importance of individual features was captured via game-theoretic Shapley values, and the results largely matched clinical intuition. Finally, we performed a post hoc analysis of the cost of clinical features to obtain optimal feature sets for a given budget constraint. The results indicate that judicious and cost-sensitive selection of clinical features can provide substantial financial and logistical savings with very little reduction in predictive performance.

## Methods

### Data description

**Figure 1:**
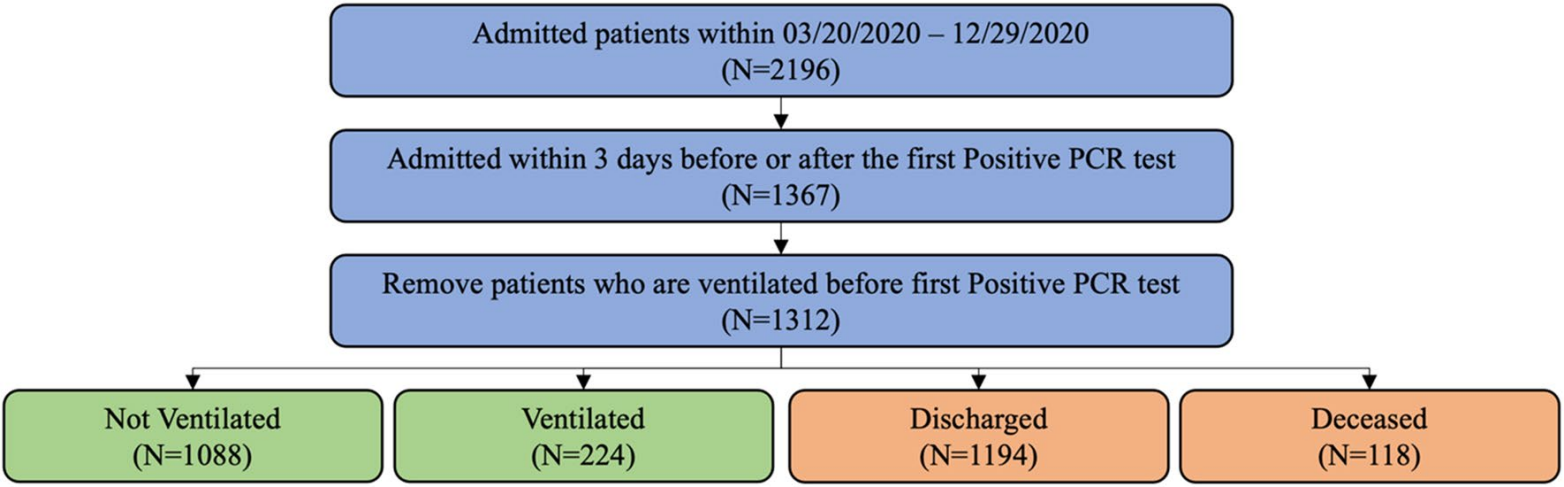
A flowchart of study enrollment. Composite ventilation related outcomes are represented by green boxes and Mortality related outcomes are represented by red boxes.

The present study was performed using EHRs from patients of ProMedica, the largest health care system in northwestern Ohio and southeastern Michigan. The EHRs were collected from patients who (1) had a positive nasopharyngeal PCR test for SARS-CoV-2 between March 20, 2020 and December 29, 2020, and (2) were admitted to the hospital within ± 3 days of the positive result. For those patients with multiple admissions in the database, only one admission that overlapped with or occurred right after the patient’s first PCR positive test was selected. Admissions where patients were put on ventilators before the first positive date were additionally filtered out. A total of 1,312 patients met these criteria. A flowchart of the study enrollment is provided in Fig. 1. The study protocol involving analysis of fully deidentified data was reviewed and approved by the respective Institutional Review Boards of ProMedica and Lawrence Livermore National Laboratory, and the study was performed in compliance with all regulations and guidelines (Expedited, Category #5 research) from the United State Department of Health and Human Services.

### Data preprocessing

Forty-nine clinical features were extracted within a ± 3 day window from the first PCR positive test date. Missing values were imputed using median imputation^20^. Continuous features were standardized using Z-score scaling (subtracting the mean and scaling to unit variance). If there were multiple observations for a feature inside the extraction window, only the observation closest to the positive test date was retained. Additionally, for the task of composite ventilation prediction, there were instances where the ± 3 day extraction window overlapped with a patient’s ventilation period. For those cases, feature values observed on or after the start of ventilation were removed.

We further noticed that clinical measurements related to oxygen levels **(**Oxygen Saturation, Inspired Oxygen Concentration, PO2, SPO2, PCO2) were highly correlated with our model’s predictions of the need for composite ventilation, even after ensuring that our extraction pipeline was sound. To make sure there could be no *information leakage*, these five oxygen measurements were removed from the task of composite ventilation prediction. As a result, 44 features were used to train our models on the task of predicting composite ventilation, whereas 49 features were used for predicting mortality. The list of features and their value ranges are presented in Tables 1 and 2 for the task of composite ventilation and mortality, respectively.

**Table 1:** Data summary of study cohort for composite ventilation.

	Ventilated (N = 224)	Not ventilated (N = 1088)	All (N = 1312)	Missing data
Age	65.0 (57.0–74.0)	62.0 (49.0–74.0)	63.0 (50.0–74.0)	0 (0%)
**Gender**^**a**^				
Male	131 (58.5%)	545 (50.1%)	676 (51.5%)	0 (0%)
Female	93 (41.5%)	543 (49.9%)	636 (48.5%)	0 (0%)
**Race**^**a**^				
Caucasians	172 (76.8%)	785 (72.2%)	957 (72.9%)	0 (0%)
African Americans	45 (20.1%)	245 (22.5%)	290 (22.1%)	0 (0%)
Others^b^	7 (3.1%)	58 (5.3%)	65 (5.0%)	0 (0%)
Social determinants of health (SDOH)^a^	190 (84.8%)	902 (75.5%)	1029 (78.4%)	0 (0%)
Pulse	92.0 (80.0–106.0)	88.0 (77.0–100.0)	88.0 (77.0–101.0)	151 (11.5%)
Respirations (breaths/min)	22.0 (20.0–26.0)	20.0 (18.0–24.0)	20.0 (18.0–24.0)	151 (11.5%)
Temperature (Celsius)	98.9 (98.1–100.35)	98.7 (98.1–99.8)	98.8 (98.1–99.9)	153 (11.7%)
Body Mass Index (BMI)	32.9 (28.2–38.8)	31.6 (26.6–37.1)	31.8 (26.8–37.3)	49 (3.7%)
Systolic blood pressure (mmHg)	127.5 (111.0–143.0)	127.0 (116.0–142.0)	127.0 (115.0–142.0)	151 (11.5%)
Diastolic blood pressure (mmHg)	71.5 (62.25–81.0)	75.0 (65.0–84.0)	74.0 (65.0–84.0)	151 (11.5%)
Pulse oximetry (%)	94.0 (92.0–96.0)	96.0 (94.0–98.0)	95.0 (93.0–98.0)	128 (9.8%)
Oxygen saturation (%)	92.0 (87.0–95.0)	93.0 (78.625–95.75)	92.6 (84.30–95.65)	1020 (77.7%)
Inspired oxygen concentration (%)	50.0 (36.0–100.0)	32.0 (21.0–40.0)	36.0 (21.0–50.0)	1083 (82.5%)
Hemoglobin (g/dL)	13.3 (12.2–14.6)	13.4 (12.0–14.6)	13.4 (12.0–14.6)	257 (19.6%)
Lymphocytes absolute (10^9^/L)	0.7 (0.5–1.0)	0.9 (0.6–1.4)	0.9 (0.6–1.3)	196 (14.9%)
Mean corpuscular hemoglobin (MCH) (pg/cell)	29.8 (28.6–31.2)	29.75 (28.3–31.2)	29.8 (28.4–31.2)	170 (13.0%)
Hematocrit (%)	39.4 (36.15–43.175)	39.6 (35.75–43.0)	39.5 (35.8–43.1)	167 (12.7%)
White blood cells (10^9^/L)	7.45 (5.1–9.8)	6.4 (4.9–8.5)	6.5 (4.925–8.7)	170 (13.0%)
Platelets (10^9^/L)	193.0 (146.0–251.0)	200.0 (157.0–261.5)	199.0 (155.0–261.0)	176 (13.4%)
Mean corpuscular hemoglobin concentration (MCHC) (g/dL)	33.7 (33.1–34.2)	33.7 (33.0–34.4)	33.7 (33.025–34.3)	170 (13.0%)
Mean Corpuscular Volume (MCV) (fL)	88.5 (85.0–92.0)	88.0 (85.0–92.0)	88.0 (85.0–92.0)	170 (13.0%)
Mean Platelet Volume (MPV) (fL)	8.7 (8.0–9.5)	8.5 (7.9–9.2)	8.5 (7.9–9.3)	177 (13.5%)
Anion gap (mmol/L)	12.0 (10.0–15.0)	11.0 (10.0–13.0)	12.0 (10.0–13.0)	187 (14.3%)
CO2 (mEq/l)	23.0 (21.0–25.0)	24.0 (22.0–26.0)	24.0 (22.0–26.0)	186 (14.2%)
PCO2 (mmHg)	34.4 (30.55–38.925)	34.0 (29.75–38.45)	34.1 (29.8–38.6)	1091 (83.2%)
PO2 (mmHg)	65.0 (58.0–80.0)	70.0 (62.0–84.0)	69.0 (59.0–83.0)	1095 (83.5%)
Sodium (mmol/L)	135.0 (132.0–138.0)	137.0 (134.0–139.0)	136.0 (134.0–139.0)	1095 (83.5%)
Chloride (mmol/L)	99.0 (96.0–102.0)	101.0 (98.0–104.0)	101.0 (97.0–104.0)	187 (14.3%)
Glucose (mg/dL)	135.0 (112.0–198.0)	119.0 (103.0–150.75)	121.0 (104.0–157.0)	187 (14.3%)
Blood Urea Nitrogen (BUN) (mg/dL)	22.0 (15.0–33.0)	17.0 (12.0–27.0)	18.0 (12.0–28.0)	186 (14.2%)
Creatinine (mg/dL)	1.12 (0.905–1.59)	0.98 (0.78–1.31)	1.0 (0.8–1.35)	184 (14.0%)
Calcium (mg/dL)	8.5 (8.2–8.8)	8.7 (8.4–9.0)	8.7 (8.4–9.0)	187 (14.3%)
Potassium Bld (mmol/L)	3.9 (3.6–4.3)	3.9 (3.6–4.2)	3.9 (3.6–4.2)	189 (14.4%)
GFR MDRD Af Amer (mL/min/1.73 m^2^)	60.0 (48.5–60.0)	60.0 (60.0–60.0)	60.0 (57.0–60.0)	207 (15.8%)
GFR MDRD Non Af Amer (mL/min/1.73 m^2^)	57.0 (40.0–60.0)	60.0 (50.0–60.0)	60.0 (47.0–60.0)	207 (15.8%)
D-Dimer (ng/mL)	349.0 (211.5–578.5)	321.5 (199.25–556.5)	331.0 (201.0–563.0)	459 (35.0%)
B-type Natriuretic Peptide (BNP) (pg/mL)	118.0 (38.0–263.0)	67.0 (27.0–183.75)	72.0 (31.0–208.0)	963 (73.4%)
Troponin (ng/mL)	0.02 (0.01–0.04)	0.01 (0.01–0.03)	0.01 (0.01–0.03)	506 (38.6%)
Procalcitonin (ng/mL)	0.24 (0.11–0.51)	0.1 (0.05–0.2)	0.11 (0.06–0.26)	433 (33.0%)
Ferritin (ng/mL)	553.0 (243.5–1017.5)	317.0 (147.0–693.0)	349.0 (160.75–737.25)	564 (43.0%)
Lactic Acid Dehydrogenase (LDH) (U/L)	340.0 (259.0–462.0)	275.0 (215.25–345.0)	284.0 (219.0–365.0)	597 (45.5%)
Sed Rate (mm/h)	59.0 (38.0–83.5)	47.0 (28.75–69.25)	49.0 (29.0–72.0)	1149 (87.6%)
C-Reactive Protein (CRP) (mg/dL)	12.35 (8.025–18.175)	6.85 (2.5–11.975)	7.9 (3.3–13.425)	532 (40.5%)
Bedside glucose (mg/dL)	162.5 (126.5–259.75)	147.0 (107.0–224.25)	151.0 (109.0–230.0)	808 (61.6%)
Chronic Kidney Disease^a,c^	34 (15.2%)	89 (8.2%)	123 (9.4%)	0 (0%)
Asthma^a,c^	13 (5.8%)	61 (5.6%)	74 (5.6%)	0 (0%)
Hypertension^a,c^	102 (45.5%)	391 (35.9%)	493 (37.6%)	0 (0%)
Diabetes^a,c^	67 (29.9%)	248 (22.8%)	315 (24.0%)	0 (0%)

Data summary of patients used for predicting Composite Ventilation. For numeric variables the median is given along with the 0.25 and 0.75 quantiles. For categorical variables the count is given along with the percentage of the population. The fourth column represents the number of missing data and their proportion to the dataset.

**Table 2:** Data summary of study cohort for Mortality.

	Deceased (N = 118)	Discharged (N = 1194)	All (N = 1312)	Missing data
Age	78.0(69.0–85.75)	61.0(49.0–72.0)	63.0 (50.0–74.0)	0 (0%)
**Gender**^**a**^				
Male	59 (50.0%)	617 (51.7%)	676 (51.5%)	0 (0%)
Female	59 (50.0%)	577 (48.3%)	636 (48.5%)	0 (0%)
**Race**^**a**^				
Caucasians	98 (83.1%)	859 (71.9%)	957 (72.9%)	0 (0%)
African Americans	19 (16.1%)	271 (22.7%)	290 (22.1%))	0 (0%)
Others^b^	1 (0.0%)	64 (0.1%)	65 (5.0%)	0 (0%)
Social determinants of health (SDOH)^a^	116 (98.3%)	976 (81.7%)	1092 (83.2%)	0 (0%)
Pulse	88.0(77.0–101.0)	88.5 (77.0–101.0)	88.0 (77.0–101.0)	140 (10.6%)
Respirations (breaths/min)	21.0(19.0–25.25)	20.0 (18.0–24.0)	20.0 (18.0–24.0)	141 (10.7%)
Temperature (Celsius)	98.6(98.1–99.35)	98.8 (98.175–99.9)	98.8 (98.1–99.9)	140 (10.6%)
Body Mass Index (BMI)	28.70 (24.475–33.925)	32.0 (27.1–37.35)	31.8 (26.8–37.3)	49 (3.7%)
Systolic blood pressure (mmHg)	131.5(113.5–144.25)	127.0 (115.0–142.0)	127.0 (115.0–142.0)	141 (10.7%)
Diastolic blood pressure (mmHg)	68.5(59.0–78.0)	75.0 (66.0–84.0)	74.0 (65.0–84.0)	141 (10.7%)
Pulse oximetry (%)	95.0(92.0–97.0)	96.0 (93.0–98.0)	88.0 (77.0–101.0)	117 (8.9%)
Oxygen saturation (%)	93.0(86.25–95.975)	93.0 (84.1–96.0)	93.0 (85.7–96.0)	972 (74.1%)
Inspired oxygen concentration (%)	70.0(40.0–100.0)	36.0 (21.0–70.0)	40.0 (28.0–90.0)	1028 (78.4%)
SPO2 (%)	94.0(91.0–96.0)	96.0 (92.0–98.0)	95.0 (92.0–97.5)	1129 (86.1%)
Hemoglobin (g/dL)	12.8(11.1–14.1)	13.4 (12.1–14.6)	13.4 (12.0–14.6)	239 (18.2%)
Lymphocytes absolute (10^9^/L)	0.8(0.5–1.3)	0.9 (0.6–1.3)	0.9 (0.6–1.3)	179 (13.6%)
Mean corpuscular hemoglobin (MCH) (pg/cell)	30.3(28.7–31.5)	29.7 (28.4–31.2)	29.8 (28.4–31.2)	154 (11.7%)
Hematocrit (%)	38.5(33.6–42.2)	39.70 (14.3–60.3)	39.6 (35.8–43.1)	151 (11.5%)
White blood cells (10^9^/L)	7.4(5.5–10.1)	6.4 (4.9–8.6)	6.5 (5.0–8.7)	154 (11.7%)
Platelets (10^9^/L)	188.5(144.0–242.0)	200.0 (156.0–261.25)	199.0 (155.0–261.0)	160 (12.2%)
Mean corpuscular hemoglobin concentration (MCHC) (g/dL)	33.4(32.6–34.2)	33.8 (33.1–34.4)	33.7 (33.0–34.3)	154 (11.7%)
Mean Corpuscular Volume (MCV) (fL)	90.0(87.0–94.0)	88.0 (85.0–92.0)	88.0 (85.0–92.0)	154 (11.7%)
Mean Platelet Volume (MPV) (fL)	8.645 (7.9–9.4)	8.5 (7.9–9.2)	8.5 (7.9–9.3)	161 (12.3%)
Anion gap (mmol/L)	13.0(10.0–14.25)	12.0(10.0–13.0)	12.0 (10.0–14.0)	171 (13.0%)
CO2 (mEq/l)	32.8(29.2–38.0)	24.00 (5.0–54.0)	24.0 (22.0–26.0)	170 (13.0%)
PCO2 (mmHg)	32.8(29.2–38.0)	24.0(22.0–26.0)	34.75 (30.0–40.1)	1038 (79.1%)
PO2 (mmHg)	68.0(56.0–91.0)	70.0(60.0–87.0)	70.0 (60.0–88.0)	1042 (79.4%)
Sodium (mmol/L)	137.0(133.75–140.0)	136.0(134.0–139.0)	136.0 (134.0–139.0)	171 (13.0%)
Chloride (mmol/L)	101.0(98.0–104.0)	101.0(97.0–104.0)	101.0 (97.0–104.0)	171 (13.03%)
Glucose (mg/dL)	132.0(108.0–178.25)	121.0(104.0–155.0)	122.0 (104.0–157.0)	171 (13.03%)
Blood Urea Nitrogen (BUN) (mg/dL)	29.0(20.0–39.5)	17.00 (3.0–228.0)	18.0 (12.0–28.0)	170 (13.0%)
Creatinine (mg/dL)	1.25(0.9175–1.7025)	17.0(12.0–27.0)	1.0 (0.8–1.37)	168 (12.8%)
Calcium (mg/dL)	8.6(8.2–8.9)	8.7(8.4–9.0)	8.7 (8.4–9.0)	171 (13.0%)
Potassium Bld (mmol/L)	4.1(3.8–4.45)	3.9(3.6–4.2)	3.9 (3.6–4.2)	173 (13.2%)
GFR MDRD Af Amer (mL/min/1.73 m^2^)	59.0(40.5–60.0)	60.0(59.0–60.0)	60.0 (56.0–60.0)	191 (14.6%)
GFR MDRD Non Af Amer (mL/min/1.73 m^2^)	49.0(33.5–60.0)	60.0(49.0–60.0)	60.0 (46.0–60.0)	191 (14.6%)
D-Dimer (ng/mL)	457.0(329.0–1104.5)	317.0(196.5–538.75)	337.0 (202.0–571.0)	435 (33.2%)
B-type Natriuretic Peptide (BNP) (pg/mL)	206.5(67.5–347.75)	68.5(28.25–169.5)	78.5 (32.0–214.0)	942 (71.8%)
Troponin (ng/mL)	0.03(0.0125–0.07)	0.01(0.01–0.03)	0.01 (0.01–0.03)	486 (37.0%)
Procalcitonin (ng/mL)	0.26(0.0975–0.5125)	0.11(0.06–0.24)	0.11 (0.06–0.26)	411 (31.3%)
Ferritin (ng/mL)	472.5(205.5–1025.25)	341.0(160.75–729.25)	354.0 (164.75–735.25)	548 (41.8%)
Lactic Acid Dehydrogenase (LDH) (U/L)	372.0(259.25–501.0)	282.0(218.0–359.0)	286.0 (220.0–370.0)	581 (44.3%)
Sed Rate (mm/h)	50.5(34.5–82.0)	49.0(29.0–71.0)	49.0 (29.0–72.0)	1143 (87.1%)
C-Reactive Protein (CRP) (mg/dL)	11.9(7.0–19.1)	7.6(3.15–13.1)	8.0 (3.3–13.725)	516 (39.3%)
Bedside glucose (mg/dL)	154.0(113.0–218.0)	151.0(109.0–233.25)	152.0 (109.0–229.0)	763 (58.2%)
Chronic Kidney Disease^a,c^	22 (18.6%)	101 (8.5%)	123 (9.4%)	0 (0%)
Asthma^a,c^	4 (3.4%)	70 (5.8%)	74 (5.6%)	0 (0%)
Hypertension^a,c^	63 (53.4%)	430 (36.0%)	493 (37.6%)	0 (0%)
Diabetes^a,c^	43 (36.4%)	272 (22.8%)	315 (24.05)	0 (0%)

Data summary of patients used for predicting Mortality. For numeric variables the median is given along with the 0.25 and 0.75 quantiles. For categorical variables the count is given along with the percentage of the population. The fourth column represents the number of missing data and their proportion to the dataset.

### Model development I: Predicting adverse outcomes without budget constraints

The following machine learning models were trained: Logistic regression, XGBoost^21^, and Gaussian process classifier (with radial basis function kernel)^22^. These models serve to establish a baseline on the predictive performance in the ideal scenario where budget is not a constraint. These models were specifically chosen because they cover a wide spectrum of ML approaches in healthcare and clinical applications. Logistic regression is the de facto technique in clinical applications because of ease of interpretation, general applicability to small datasets, and availability in statistical software packages^23, 24^. A limitation of logistic regression is the underlying assumption of linearity^25^, which is often too strong for many clinical tasks. Gaussian process classifiers remove the assumption of linearity while performing well on small datasets, but they are less interpretable than logistic regression. Among non-linear classifiers, Gaussian process classifier is arguably the most powerful technique with sound statistical properties. Additionally, it is possible to train this model in the presence of sparse and missing data, which makes it an ideal choice for analyzing EHRs. The Gaussian process classifier has been applied to various detection and prediction tasks, including early recognition of sepsis^26^, in-hospital mortality prediction for preterm infants^27^, and health monitoring with wearable sensors^28^. XGBoost is a relatively new ensemble machine learning model, and it represents the best-in-class among tree-based classifiers, combining strong predictive performance and the interpretability of decision trees. In recent literature, XGBoost has often been shown to provide improved predictive performance in a wide range of clinical applications. Sharma et al.^29^ use XGBoost for diagnosing depression in unbalanced datasets. Chang et al.^30^ apply XGBoost to the task of predicting hypertension outcomes, and they find that this model has better predictive performance than a random forest or a support vector machine.

Models were trained and evaluated following fivefold train/test splits to account for variances, resulting in 80%/20% training and testing splits. For each train/test split, model hyperparameters were optimized by performing fourfold cross validation on the training set, leading to 80%/20% splits into optimization and validation sets—that is, the optimization and validation sets contain 64% and 16% of the full dataset. The Optuna optimization framework^31^ was used for hyperparameter tuning with the objective of maximizing AP. Additionally, a post-hoc feature importance analysis was performed by applying the SHAP framework^11^, a game-theoretic feature attribution framework that reveals how each feature per sample contributes to the decisions made by the model for the corresponding sample. All of our experiments were conducted using Python 3.8, and models were implemented in the Scikit-Learn framework^32^.

### Model development II: Feature selection under budget constraints

In this section, the financial costs of the clinical features were taken into consideration when training predictive models. For a pre-defined cost structure, the goal was to find the set of clinical features that provides the highest predictive performance in terms of AP. One could find the best subset of clinical features iteratively by trying all possible combinations of features obeying the budget constraint; however, this method would be computationally expensive, as it scales exponentially with the number of features. Therefore, we propose an alternative selection scheme. Intuitively, several clinical features are often recorded collectively and can be grouped together. For example, temperature, blood pressure, and pulse are often measured together when the patient is first admitted to the hospital. Guided by clinical experts, 11 groups of clinical features and their relative collection costs were defined (Table 3). By training machine learning models on each combination of groups, we only must explore 2^11^ combinations rather than 2^44^ combinations for predicting ventilation and 2^49^ for predicting mortality. As XGBoost was the best performing model without budget constraints, it was also selected for the budget-constrained prediction task. XGBoost was trained for all 2^11^ combinations of feature groups to obtain the combination with highest AP for a given budget constraint.

**Table 3:** Cost structure of clinical features.

Group	Cost
DCM (Demographic and comorbidities: Age, Sex, Gender, Race, Respiratory Rate, BMI, Temperature, Systolic/Diastolic Blood Pressure, Pulse Oximetry, FIO2, O2 Sat, SPO2, Asthma, Chronic Kidney Disease, Diabetes, Hypertension)	0
BMP (Basic Metabolic Profile: Sodium, Chloride, Glucose, GFR MDRD Non Af Amer, Creatinine, BUN, Calcium, Potassium, Bld)	1
CBC (Complete Blood Count: Hemoglobin, Lymphocyte, MCH, Hematocrit, White Blood Cells, Platelets, MCHC, MCV, MPV)	1
D-dimer	2
LDH	2
Sed rate	2
CRP	2
BNP	3
Troponin	3
Procalcitonin	3
Ferritin	3

Pre-defined cost structure for each group of features (may be modified based on the healthcare facility) based on a relative cost score where 0 indicates clinical features that are least expensive and 3 indicates clinical features that are most expensive.

### Informed consent

The study protocol involving analysis of fully de-identified data was reviewed and approved with Full Waiver of informed consent granted (Expedited, Category #5 research) by the respective Institutional Review Board's of ProMedica and Lawrence Livermore National Laboratory. The study was performed in compliance with all regulations and guidelines from the United State Department of Health and Human Services.

## Results

We report results on five test sets (which together span the entire dataset), and provide the mean and standard deviation of AP and AUC scores. The proposed ML framework achieved the best mean (standard deviation) across validation sets with AUC of 0.723 (0.038) and AP of 0.379 (0.038) for composite ventilation and AUC of 0.802 (0.029) and AP of 0.354 (0.065) for mortality. Note, for comparison, a classifier that completely disregards the data can achieve an AUC of 0.5 and AP of 0.090 (for composite ventilation) and 0.171 (for mortality). A distinction between our work and others is that we chose the hyperparameters of our models by optimizing AP rather than AUC. Our motivation is that AP is a more meaningful measure of performance in the presence of significant class imbalance. This is predominantly true in our case, where the number of deceased patients (N = 224) as well as the number of patients who required composite ventilation (N = 118) is a small fraction of the total cohort (N = 1312).

**Figure 2:**
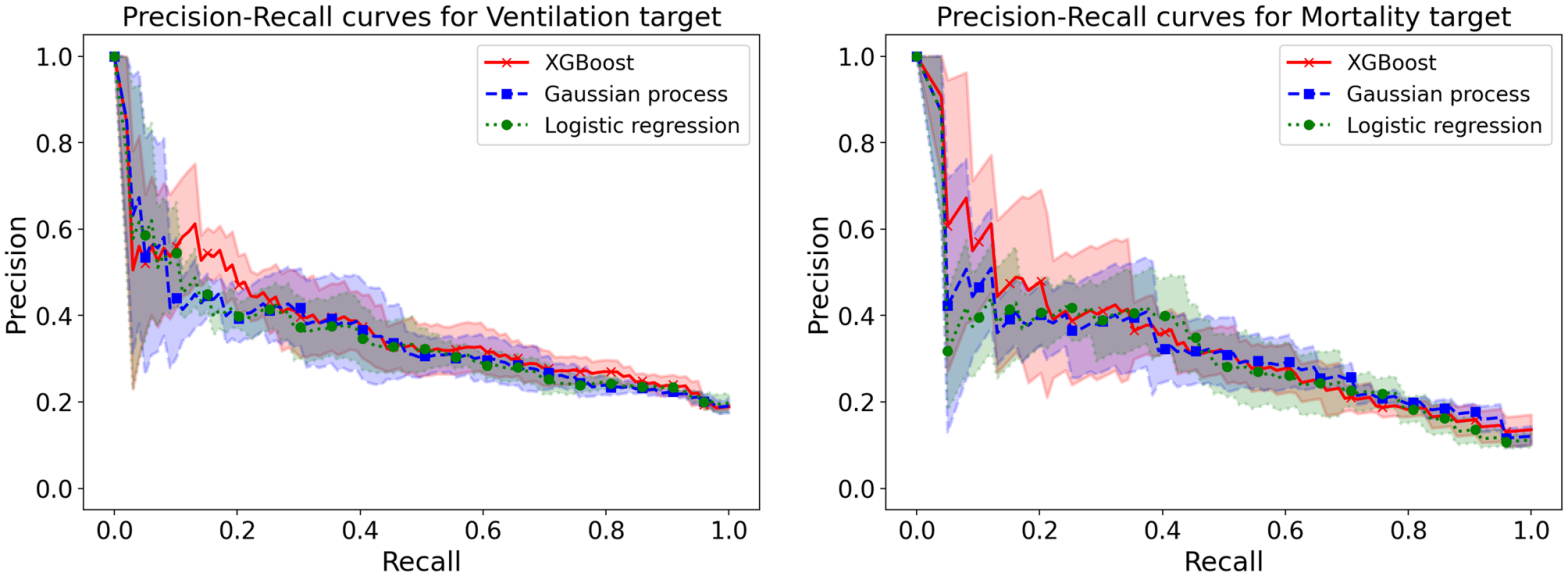
Precision-Recall (PR) curves on the ventilation and mortality tasks. The lines are the mean PR curves over 5 different train/test splits and the shaded areas represent ± 1 standard deviations from the means. In both tasks, the XGBoost model has the best PR curve overall, which is reflected in its average precision (AP) score in Table 4.

**Figure 3:**
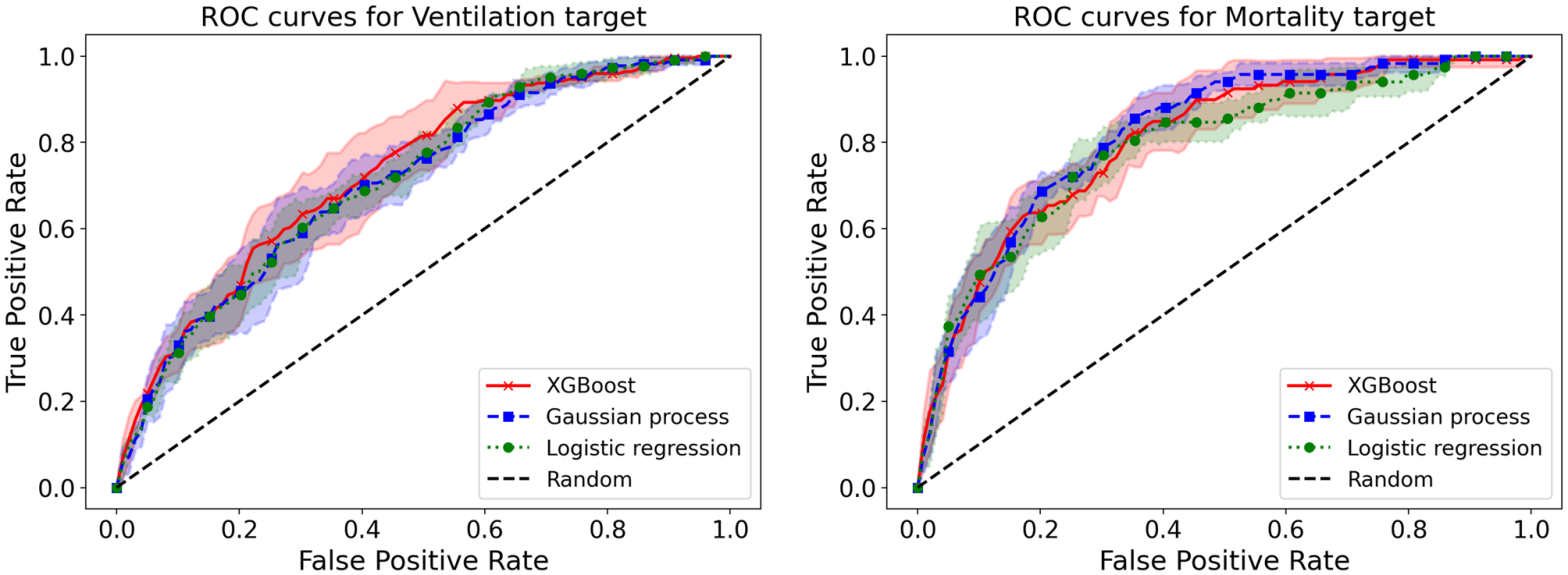
Receiver operating characteristic (ROC) curves on the ventilation and mortality tasks. The lines are the mean ROC curves over 5 different train/test splits and the shaded areas represent ± 1 standard deviations from the means. The performance of the three models is comparable, with XGBoost having the best performance in the ventilation task and the Gaussian process classifier having slightly better performance in the mortality task. The area under the curve (AUC) scores for all the models are reported in Table 4.

**Figure 4:**
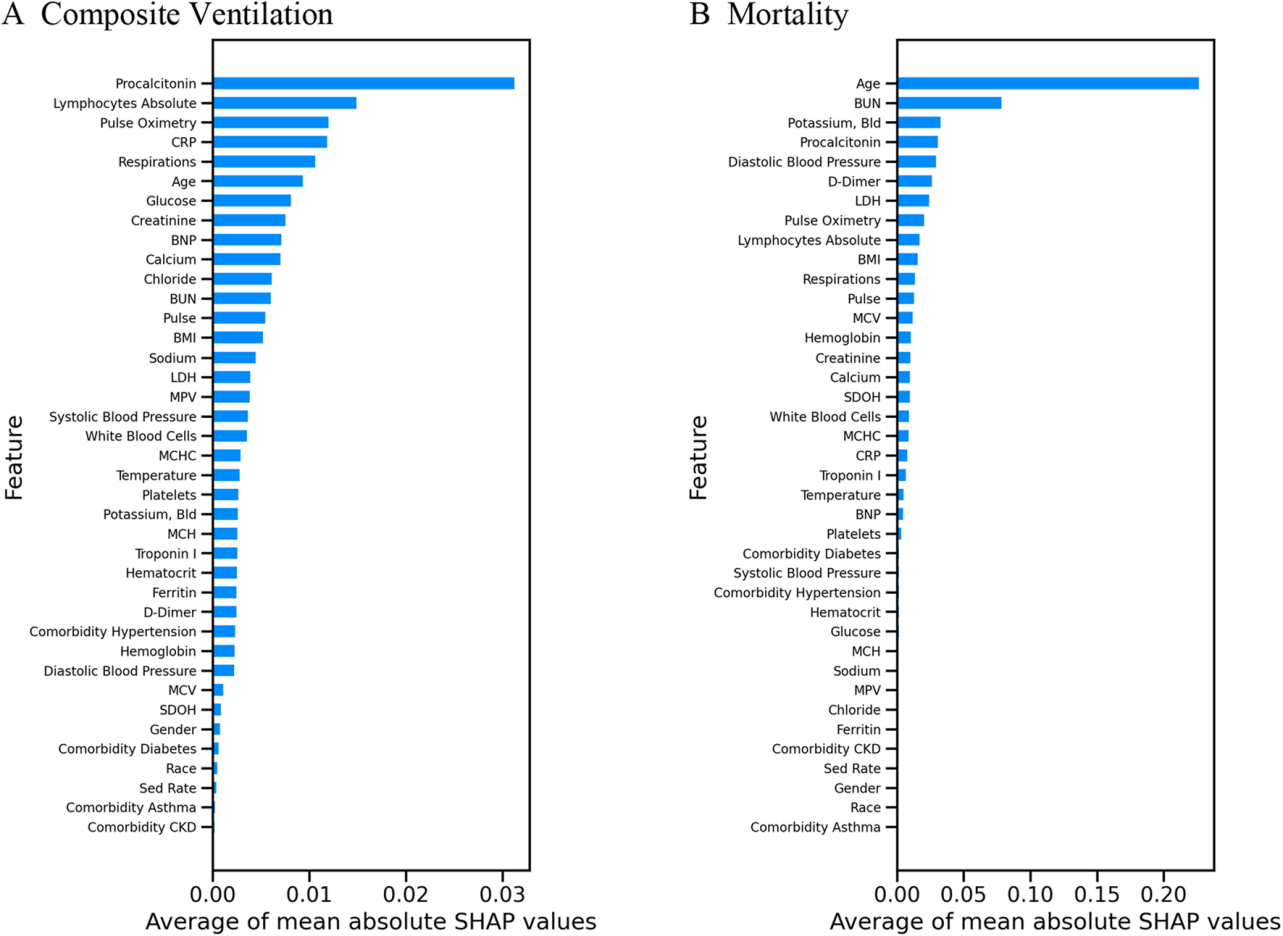
Feature importance of a trained XGBoost model. The higher the absolute SHAP value, the greater the contribution of the feature to the predicted outcome. The SHAP values are averaged over 5 folds of test splits that span the whole dataset. (**A**) The top three most important features for predicting ventilation are Procalcitonin, Lymphocytes Absolute and Pulse Oximetry. (**B**) The top three most important features for predicting mortality are Age, BUN and Potassium.

The results of logistic regression, XGBoost, and Gaussian process classifier without budget constraints are provided in Table 4. We also provide the Receiver Operating Characteristic (ROC) curves and Precision–Recall curves for the three ML approaches (Figs. 2 and 3). Among the three classifiers, for both adverse outcomes (composite ventilation and mortality), XGBoost provides the highest AP (as well as the highest AUC for composite ventilation and the second highest AUC for mortality). Feature importance for XGBoost—computed using the SHAP framework—for both outcomes are shown in Fig. 4. For predicting ventilation, the five most important features (when averaged over five test sets) were *procalcitonin*, *lymphocyte count*, *pulse oximetry*, *C-Reactive Protein (CRP)*, and *age*. Similarly, for predicting mortality, the top five features were *age*, *blood urea nitrogen (BUN)*, *serum potassium*, *diastolic blood pressure*, and *D- Dimer levels*.

**Table 4:** Prediction performance using all clinical features.

Model	Outcome	AP mean (std)	AUC mean (std)
XGBoost	Composite ventilation	0.379 (0.038)	0.723 (0.038)
Gaussian process	Composite ventilation	0.357 (0.037)	0.713 (0.023)
Logistic regression	Composite ventilation	0.361 (0.035)	0.717 (0.013)
XGBoost	Mortality	0.354 (0.065)	0.802 (0.029)
Gaussian process	Mortality	0.338 (0.055)	0.819 (0.012)
Logistic regression	Mortality	0.328 (0.067)	0.805 (0.029)

Adverse outcome prediction performance at the point of entry for the various ML models in terms of average precision (AP) and area under receiver operating curve (AUC).

The optimal sets of features for predicting composite ventilation under different budget constraints are reported in Table 5. Since Demographics and Comorbidities (DCM) are readily available upon a patient’s admission, this information was considered to be free of cost and included in every feature set. Under different budget constraints, the model with highest mean AP across cross-validated test sets was selected. As the available budget increased from 0 to 15, the AP increased from a mean (standard deviation) of 0.289 (0.016) to 0.382 (0.047). The performance peaked at a budget level of 15, the optimal set of features being DCM, BMP, D-dimer, LDH, Sedrate, CRP, BNP, and Procalcitonin. Increasing the budget (i.e., adding more features) beyond this point did not yield a better performance.

**Table 5:** Optimal set of features at different budget levels for predicting composite ventilation.

Budget	Total cost	Optimal set of features	AP mean (std)
1	1	DCM, BMP	0.289 (0.016)
2	2	DCM, CRP	0.314 (0.071)
3	3	DCM, BMP, LDH	0.331 (0.034)
4	4	DCM, BMP, CBC, CRP	0.353 (0.045)
5	4	DCM, BMP, CBC, CRP	0.353 (0.045)
6	4	DCM, BMP, CBC, CRP	0.353 (0.045)
7	7	DCM, BMP, CBC, D-dimer, Procalcitonin	0.363 (0.051)
8	7	DCM, BMP, CBC, D-dimer, Procalcitonin	0.363 (0.051)
9	7	DCM, BMP, CBC, D-dimer, Procalcitonin	0.363 (0.051)
10	10	DCM, BMP, CBC, CRP, BNP, Troponin	0.371 (0.03)
11	10	DCM, BMP, CBC, CRP, BNP, Troponin	0.371 (0.03)
12	10	DCM, BMP, CBC, CRP, BNP, Troponin	0.371 (0.03)
13	10	DCM, BMP, CBC, CRP, BNP, Troponin	0.371 (0.03)
14	10	DCM, BMP, CBC, CRP, BNP, Troponin	0.371 (0.03)
15	15	DCM, BMP, D-dimer, LDH, Sedrate, CRP, BNP, Procalcitonin	0.382 (0.047)
16	15	DCM, BMP, D-dimer, LDH, Sedrate, CRP, BNP, Procalcitonin	0.382 (0.047)
17	15	DCM, BMP, D-dimer, LDH, Sedrate, CRP, BNP, Procalcitonin	0.382 (0.047)
18	15	DCM, BMP, D-dimer, LDH, Sedrate, CRP, BNP, Procalcitonin	0.382 (0.047)
19	15	DCM, BMP, D-dimer, LDH, Sedrate, CRP, BNP, Procalcitonin	0.382 (0.047)
20	15	DCM, BMP, D-dimer, LDH, Sedrate, CRP, BNP, Procalcitonin	0.382 (0.047)
21	15	DCM, BMP, D-dimer, LDH, Sedrate, CRP, BNP, Procalcitonin	0.382 (0.047)
22	15	DCM, BMP, D-dimer, LDH, Sedrate, CRP, BNP, Procalcitonin	0.382 (0.047)

Total cost is the sum of the cost of each feature group in the selected set.

The optimal set of features for predicting mortality under different budget constraints are reported in Table 6. Under different budget constraints, the model with highest mean AP across cross-validated test sets was selected. As the available budget increased from 0 to 17, the AP increased from 0.285 (0.048) to 0.355 (0.075). The performance peaked at a budget constraint of 17, the optimal set of features being DCM, BMP, CBC, D-dimer, LDH, CRP, BNP, Procalcitonin and Ferritin. Increasing the budget beyond this point did not yield a better performance.

**Table 6:** Optimal set of features at different budget levels for predicting mortality.

Budget	Total cost	Optimal set of features	AP mean (std)
1	1	DCM, BMP	0.285 (0.048)
2	2	DCM, LDH	0.311 (0.06)
3	3	DCM, BMP, LDH	0.317 (0.05)
4	4	DCM, LDH, CRP	0.34 (0.07)
5	4	DCM, LDH, CRP	0.34 (0.07)
6	4	DCM, LDH, CRP	0.34 (0.07)
7	4	DCM, LDH, CRP	0.34 (0.07)
8	8	DCM, BMP, LDH, CRP, Troponin	0.343 (0.079)
9	8	DCM, BMP, LDH, CRP, Troponin	0.343 (0.079)
10	10	DCM, BMP, LDH, Sedrate, CRP, Procalcitonin	0.351 (0.08)
11	10	DCM, BMP, LDH, Sedrate, CRP, Procalcitonin	0.351 (0.08)
12	10	DCM, BMP, LDH, Sedrate, CRP, Procalcitonin	0.351 (0.08)
13	10	DCM, BMP, LDH, Sedrate, CRP, Procalcitonin	0.351 (0.08)
14	10	DCM, BMP, LDH, Sedrate, CRP, Procalcitonin	0.351 (0.08)
15	10	DCM, BMP, LDH, Sedrate, CRP, Procalcitonin	0.351 (0.08)
16	10	DCM, BMP, LDH, Sedrate, CRP, Procalcitonin	0.351 (0.08)
17	17	DCM, BMP, CBC, D-dimer, LDH, CRP, BNP, Procalcitonin, Ferritin	0.355 (0.075)
18	17	DCM, BMP, CBC, D-dimer, LDH, CRP, BNP, Procalcitonin, Ferritin	0.355 (0.075)
19	17	DCM, BMP, CBC, D-dimer, LDH, CRP, BNP, Procalcitonin, Ferritin	0.355 (0.075)

Total cost is the sum of the cost of each feature group in the selected set.

## Discussion

Several recent publications have also proposed using XGBoost or very similar models to predict mortality or ventilation using EHR^3, 5–7^. For mortality prediction, Yan et al.^3^ identified LDH, Lymphocytes, and CRP as the most significant features. Using a cohort in New York City, NY, USA, Yadaw et al.^4^ compared different models including logistic regression, support vector machine (SVM), random forest (RF), and XGBoost for mortality prediction and identified the most informative features as Age, Oxygen Saturation, and the type of visit (tele-health or inpatient/outpatient). Bertsimas et al.^12^ analyzed cohorts in Spain, Greece, and USA. The authors used the SHAP framework in their study, and they reported that BUN, CRP, and Oxygen Saturation were the most informative features for mortality.

**Figure 5:**
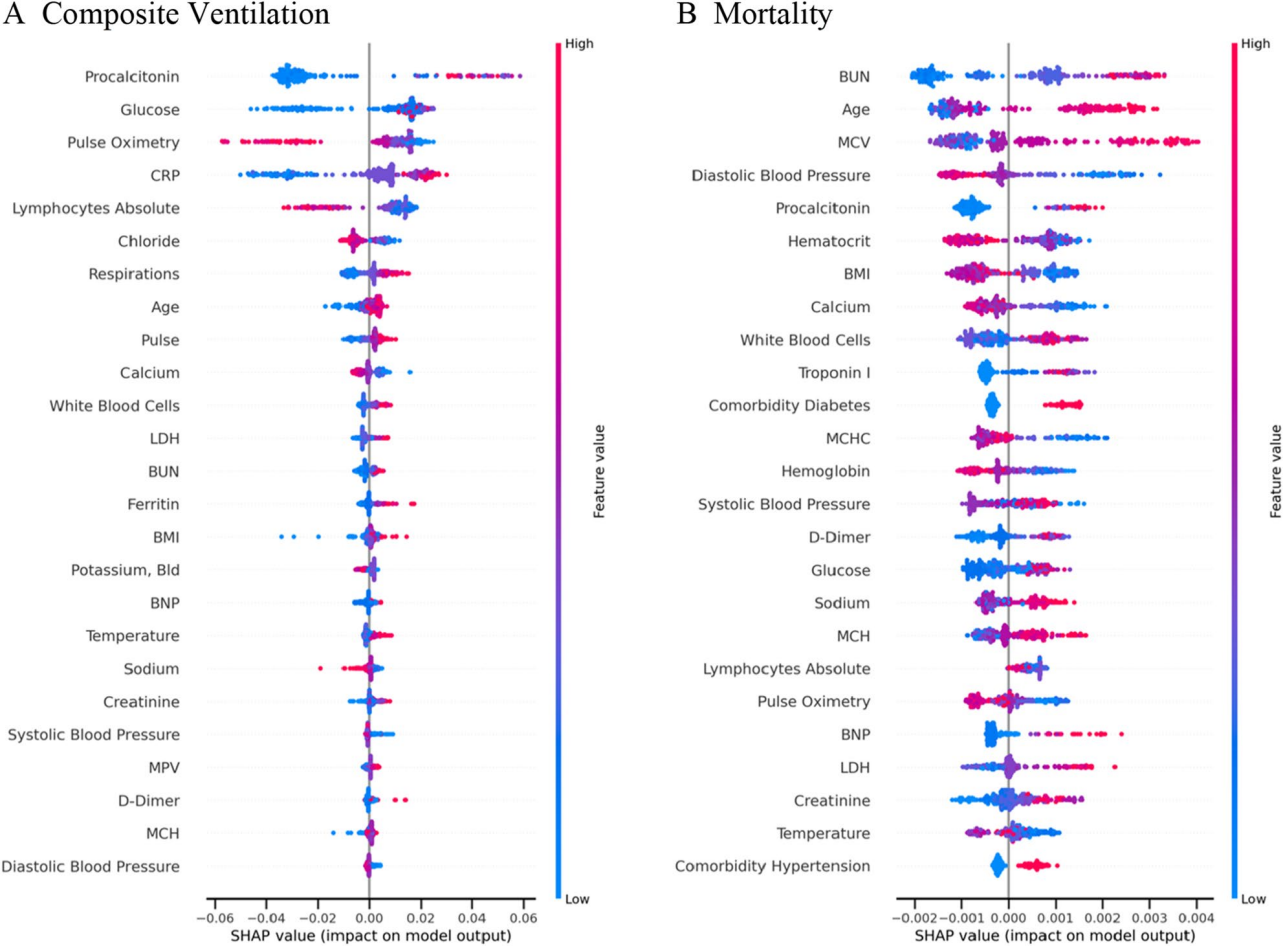
Scatter plots of SHAP values versus unnormalized values for selected features. (**A**–**C**) The top three most significant features for predicting composite ventilation for the best performing fold. (**D**–**F**) The top three most significant features for predicting mortality for the best performing fold.

**Figure 6:**
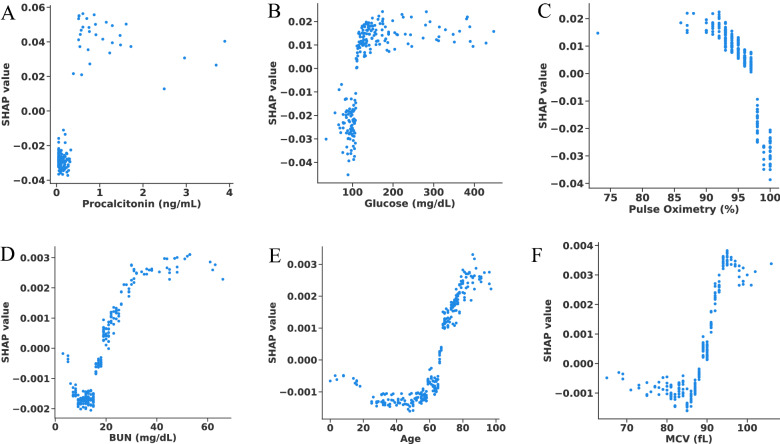
SHAP scatter plot for prediction using all features for the best performing fold. Positive SHAP values imply the corresponding feature was indicative of ventilation/death. Negative SHAP values imply the corresponding feature was indicative of no ventilation/discharged. A zero SHAP value implies the feature has no impact on the predicted outcome. The normalized range of value of features are color-coded.

After training and testing a model using all available clinical features, we found the top contributing factors for predicting adverse outcomes. To do so, we applied the SHAP framework^11^ to the best performing model (XGBoost), selected the top performing fold, and plotted the relationship between feature values and feature significance (Figs. 5, 6). Note that higher Shapley values are indicative of the adverse outcome (composite ventilation or death) and vice versa. In general, the Shapley plots uncovered relationships between clinical features and outcomes which are well supported by existing clinical literature. For example, lower values of Procalcitonin reduce the likelihood of being labelled as requiring ventilation. Aligned with other studies^33–35^, our analysis suggests that Procalcitonin can be a robust lab test for predicting ventilation. In addition to Procalcitonin, Pulse Oximetry and Absolute Lymphocyte Count exhibit a clear relationship to ventilation, where lower levels of Pulse Oximetry and Absolute Lymphocytes reduce the chances of being labelled as requiring ventilation. Our findings demonstrate that, used in combination, these markers are highly predictive of composite ventilation.

From a clinical point of view, our study suggests that the most relevant set of variables that predicted ventilation as an outcome were elevated Procalcitonin levels, elevated Lymphocyte Count, lower Pulse Oximeter readings, elevated CRP, and older age (based on averaged SHAP values across all test splits, Fig. 4 (a)). The study also suggests that the variables that best predicted mortality as the outcome were older age, elevated BUN, elevated serum potassium, elevated diastolic blood pressure, and elevated D-dimer levels (Fig 4 (b)). This study was not designed to evaluate causality for the clinical data but suggests that these clinical data serve as appropriate surrogate markers for the underlying biological causes of respiratory failure and death in infection with SARS-CoV-2.

Regarding composite ventilation, from a pathophysiologic perspective, the patients that have an elevated procalcitonin level and elevated lymphocytic count are more likely to have bacterial and/or viral superinfection, which would be associated with increased risk of requiring ventilation. The lower pulse oximeter readings and elevated levels of inflammation seen with the elevated CRP reflect an increased severity of pulmonary damage that would be seen in patients progressing to ventilation. Extremes of age are well-recognized risk factors for adverse outcomes in the majority of severe medical conditions, and COVID-19 is no exception^36, 37^.

Regarding mortality, elevated BUN and serum potassium are reflections of impaired renal function, and acute kidney injury is associated with increased hospital mortality in all studies of sepsis^37–40^. The mechanism by which COVID-19 impacts renal function remains unclear: direct cytotoxic effects, cytokine storm related hypotension, renal hypoxia secondary to systemic hypoxia, and micro-coagulation in the renal vasculature (which may also be reflected in an elevated D-dimer level) have all been proposed as potential mechanisms^41–43^. Regarding elevated diastolic blood pressure as a marker for increased risk of mortality, this may be a surrogate marker for cardiac disease instead of an independent pathophysiologic mechanism.

Though not among the top predictors for mortality, mean corpuscular volume (MCV) is an interesting marker that has been correlated with worse outcomes both at lower values^44^ and higher values^45^. In our study population, non-alcoholic fatty liver disease is common and may be unrecognized given that mean BMI was high and fatty liver disease is often seen in obesity, whereas other studies demonstrating a low MCV being predictive of worse outcomes have been done in areas of the world where ß-thalassemia is more common. This indicates that MCV needs to be considered in the context of other population factors.

However, contrary to most recent publications^46^, Fig. 5b shows that higher BMI *decreased* the likelihood of being labelled as deceased. Higher BMI being associated with better outcomes has also been reported in veterans^47^. This may be attributed to *collider bias,* as the number of obese people outnumber the non-obese people in our cohort and obese people are also more likely to be hospitalized for COVID-19.

**Figure 7:**
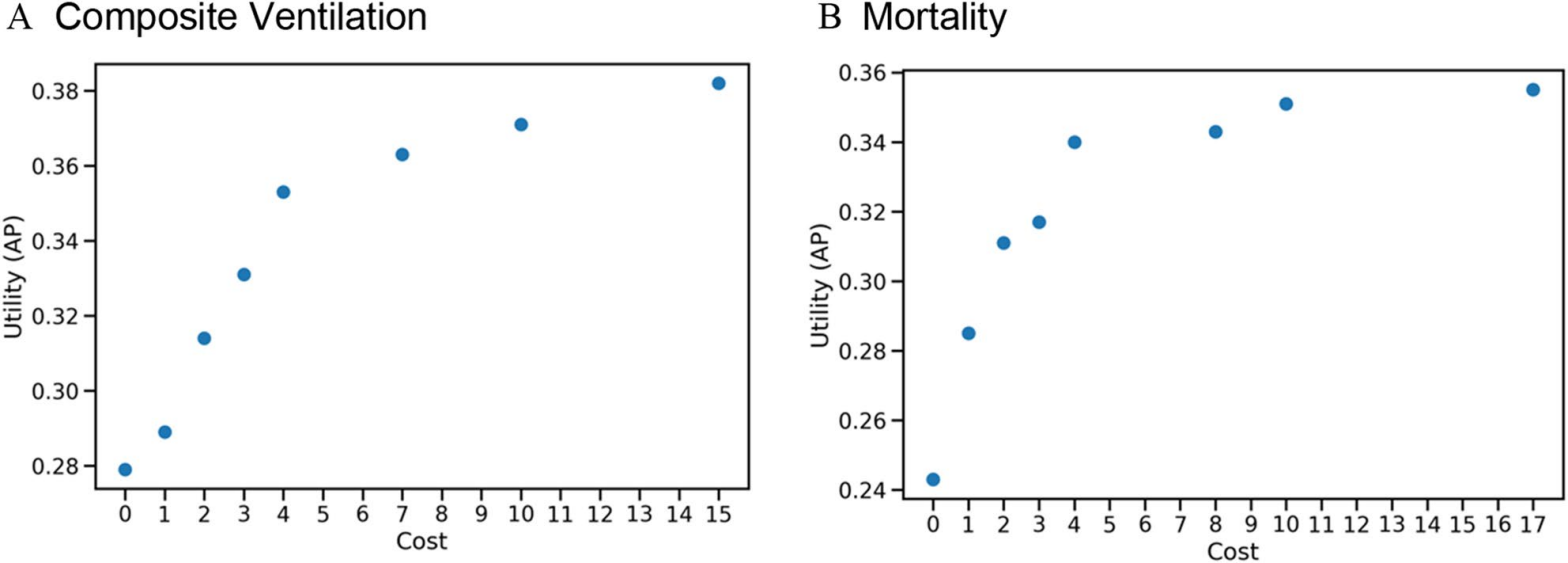
Visualization of Total cost versus Utility. Although an increase in budget allows more clinical feature groups in our selection, this does not guarantee an increase in performance. The performance maximizes when the total costs of features is 15 for composite ventilation and 17 for mortality, and any additional feature does not increase utility.

The major focus of this work is the post-hoc analysis of models by incorporating budget constraints into the problem formulation. A notable trend in both mortality and ventilation prediction is that the best predictive performance is achieved for a significantly smaller set of *optimal features*. In general, predictive performance increases with allowable budget, but there is a clear point of inflexion for both composite ventilation and mortality (Fig. 7). For composite ventilation, the predictive performance is at 92% of the maximum AP at 18% of the maximum budget. Similarly, for mortality, the predictive performance is at 96% of the maximum AP at 18% of the maximum budget. This implies that when it comes to making healthcare affordable, clinicians can choose a smaller set of features that not only fits within their budget but also achieves close-to-optimal performance. For example, when predicting ventilation (Table 5 and Fig. 7a) we can achieve a 43% reduction in cost with only a 3% reduction in performance by using the feature set DCM, BMP, CBC, and CRP rather than the optimal feature set DCM, BMP, CBC, CRP, D-dimer, and Procalcitonin. Similarly, when predicting mortality (Table 6 and Fig. 7b) we can achieve a 50% reduction in cost with only a 1% reduction in performance by using the feature set DCM, LDH, and CRP rather than the optimal feature set DCM, BMP, LDH, CRP, and Troponin. This substantial drop in cost may be attributed to the fact that Troponin and Procalcitonin are expensive lab tests. Overall, we provide a tool to perform cost–benefit analysis for selecting the most efficient and cost-effective lab tests for prediction of adverse outcomes.

## Limitations

As with most retrospective cohort outcomes studies, an inherent limitation is the associative nature of the study analysis and the fact that disease prediction markers used in the modeling may be correlated. Thus, a better approach to selecting clinical features and prediction markers may be network analysis-based node selection, which considers the clinical and biological connections between markers and reduces the redundancy^48, 49^. Additionally, future studies may incorporate clinical markers that are reported to be associated with outcomes of COVID-19 in meta-analysis with large-scale cohorts^50, 51^ into the training of the prediction model. Indeed, a limitation of our present study is the lack of validation on another independent dataset of hospitalized COVID-19 patients, due to limited dataset availability. Furthermore, the potential causal effects of clinical variables on the adverse outcomes for COVID-19 patients could be further interrogated through causal inference analysis methods, such as mendelian randomization analysis^52–54^, which may help understanding clinical and biological mechanisms of our prediction models.

## Conclusion

The current study provides a point-of-care model that allows improved resource allocation and evidence-based management that can be applied to improve patient care in the setting of COVID-19. We present a machine learning framework to evaluate risk of clinical deterioration for patients with SARS-CoV-2 infection at the time of medical evaluation and to determine appropriate medical management for newly admitted patients. The proposed approach also identifies the top clinical factors predictive of an adverse outcome and integrates budget considerations in the prediction framework. More specifically, based on post-hoc optimization, the approach identifies the optimal set of clinical variables for a pre-defined budget constraint. The proposed framework has the potential for real-life impact by helping clinicians make fast and reliable decisions regarding patient risk stratification in a cost-effective manner. Incorporating financial considerations into data-driven prediction models can be beneficial to cost-constrained healthcare systems where costly laboratory tests may not be always readily available.
